# Flexible trial design in practice - stopping arms for lack-of-benefit and adding research arms mid-trial in STAMPEDE: a multi-arm multi-stage randomized controlled trial

**DOI:** 10.1186/1745-6215-13-168

**Published:** 2012-09-15

**Authors:** Matthew R Sydes, Mahesh KB Parmar, Malcolm D Mason, Noel W Clarke, Claire Amos, John Anderson, Johann de Bono, David P Dearnaley, John Dwyer, Charlene Green, Gordana Jovic, Alastair WS Ritchie, J Martin Russell, Karen Sanders, George Thalmann, Nicholas D James

**Affiliations:** 1 MRC Clinical Trials Unit, London, UK; 2 School of Medicine, Cardiff University, Cardiff, UK; 3 The Christie and Salford Royal Hospitals Foundations Trusts, Manchester, UK; 4 The Royal Hallamshire Hospital, Sheffield, UK; 5 Institute of Cancer Research and Royal Marsden Hospitals Foundation Trust, Sutton, UK; 6 Prostate Cancer Support Federation, Stockport, UK; 7 Beatson West of Scotland Cancer Centre, Glasgow, UK; 8 Inselspital, Bern, Switzerland; 9 School of Cancer Sciences, University of Birmingham, Birmingham, UK

**Keywords:** Novel design, Multi-arm multi-stage design, Implementation, Prostate cancer, Methodology, Randomized controlled trial

## Abstract

**Background:**

Systemic Therapy for Advanced or Metastatic Prostate cancer: Evaluation of Drug Efficacy (STAMPEDE) is a randomized controlled trial that follows a novel multi-arm, multi-stage (MAMS) design. We describe methodological and practical issues arising with (1) stopping recruitment to research arms following a pre-planned intermediate analysis and (2) adding a new research arm during the trial.

**Methods:**

STAMPEDE recruits men who have locally advanced or metastatic prostate cancer who are starting standard long-term hormone therapy. Originally there were five research and one control arms, each undergoing a pilot stage (focus: safety, feasibility), three intermediate ‘activity’ stages (focus: failure-free survival), and a final ‘efficacy’ stage (focus: overall survival). Lack-of-sufficient-activity guidelines support the pairwise interim comparisons of each research arm against the control arm; these pre-defined activity cut-off becomes increasingly stringent over the stages. Accrual of further patients continues to the control arm and to those research arms showing activity and an acceptable safety profile. The design facilitates adding new research arms should sufficiently interesting agents emerge. These new arms are compared only to contemporaneously recruited control arm patients using the same intermediate guidelines in a time-delayed manner. The addition of new research arms is subject to adequate recruitment rates to support the overall trial aims.

**Results:**

(1) Stopping Existing Therapy: After the second intermediate activity analysis, recruitment was discontinued to two research arms for lack-of-sufficient activity. Detailed preparations meant that changes were implemented swiftly at 100 international centers and recruitment continued seamlessly into Activity Stage III with 3 remaining research arms and the control arm. Further regulatory and ethical approvals were not required because this was already included in the initial trial design.

(2) Adding New Therapy: An application to add a new research arm was approved by the funder, (who also organized peer review), industrial partner and regulatory and ethical bodies. This was all done in advance of any decision to stop current therapies.

**Conclusions:**

The STAMPEDE experience shows that recruitment to a MAMS trial and mid-flow changes its design are achievable with good planning. This benefits patients and the scientific community as research treatments are evaluated in a more efficient and cost-effective manner.

**Trial registration:**

ISRCTN78818544, NCT00268476

First patient into trial: 17 October 2005

First patient into abiraterone comparison: 15 November 2011

## Background

### Multi-arm multi-stage trials

Adaptive clinical trials are increasingly discussed and presented. The multi-arm, multi-stage (MAMS) design [1,2] is one example of a flexible, seamless phase II/III randomized controlled trial. Detail on the general rationale for the MAMS design has been published elsewhere [3], but in brief, this approach allows for several research approaches to be assessed simultaneously against a common control group. Accrual resources are directed away from arms that show either insufficient activity on an intermediate primary outcome measure or unacceptable toxicity so that recruitment becomes increasingly focused towards the more promising research arms and the control arm. The MAMS design provides an efficient method for acquiring multi-agent prospective randomized data synchronously, requiring less time and needing fewer patients while simultaneously reducing the trial bureaucracy that is associated with a program of separate Phase II and Phase III trials. The MAMS approach thereby increases the likelihood of identifying one or more successful treatments in a single trial. It also decreases the likelihood of the whole trial stopping prematurely because the chance of multiple treatment approaches being insufficiently tolerated or ineffective is reduced.

The ability to add new research arms is an added advantage; initiating and conducting clinical trials remains resource-intensive and time-consuming both in terms of securing funding and gaining regulatory approval. The concept of adding new research arms within the context of an ongoing trial, thereby utilizing its existing machinery and recruitment base, is appealing. This can bring efficiencies in cost, personnel, and recruitment, leading to a reduction by many years in the time taken to initiate and recruit to the trial. This could further allow companies a longer period to generate profits within their patent.

### The STAMPEDE trial

**Figure 1:**
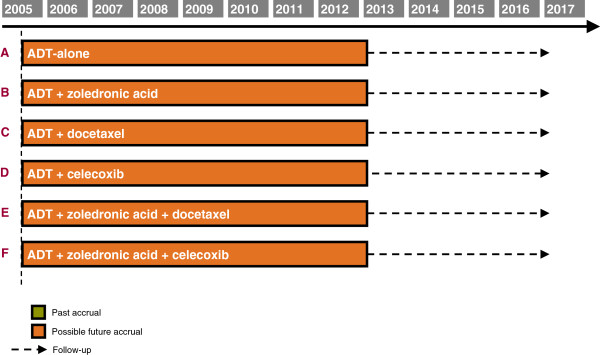
Trial arms open to allocation and further timelines at start of trial.

**Figure 2:**
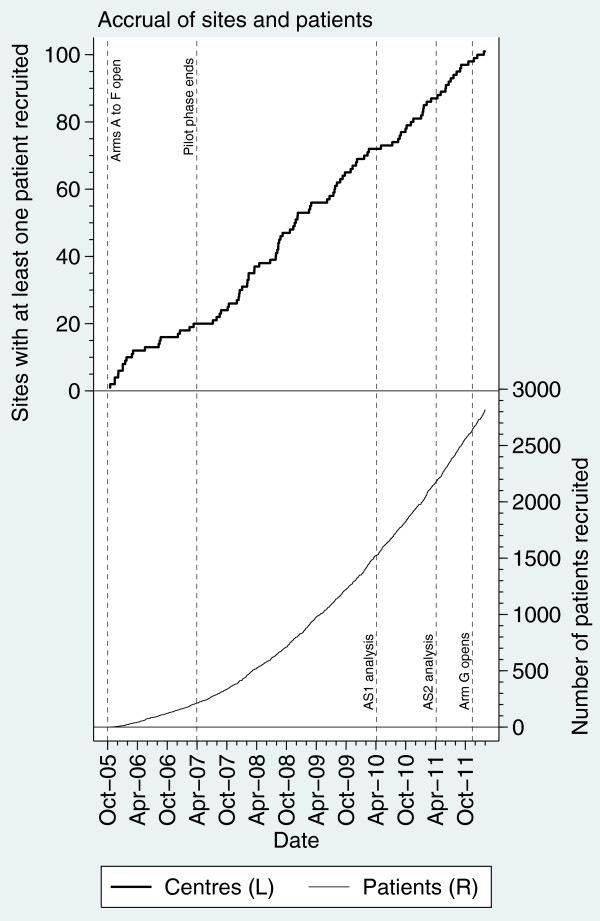
Cumulative accrual of patients and activation of sites.

STAMPEDE (Systemic Therapies for Advancing or Metastatic Prostate cancer: Evaluation of Drug Efficacy) is a multi-center, open-label, multi-arm multi-stage randomized trial. The aim is to identify new treatments that could improve survival in men with high-risk locally advanced or metastatic prostate cancer and who are starting standard care with long-term hormone therapy (HT) with androgen deprivation. STAMPEDE assesses many research treatments, all added to standard HT: docetaxel, zoledronic acid, celecoxib and, now, abiraterone. The detailed clinical rationale of the trial has been presented elsewhere [4-6]. The trial is approved by the appropriate ethics and regulatory authorities. Figure 1 depicts the original trial and the anticipated timelines; Figure 2 shows observed accrual rates.

### Rationale for publication

The methodological details of STAMPEDE’s design and the practical issues of initiating the trial have been published elsewhere [7], but here we report on ongoing trial logistics, discussing the early stopping of two trial arms after an intermediate lack-of-sufficient-activity analysis (often referred to as a ‘lack-of-benefit’ for short) and the activation of a new research arm.

## Managing early stopping following lack-of-benefit analysis

### Trial design

The multi-arm multi-stage design of STAMPEDE has a series of formal intermediate analyses for each trial arm that take place at the end of each intermediate activity stage (AS); STAMPEDE has three such stages. Each research arm is compared separately to the common control arm in a pairwise fashion. However, there is no comparison of the research arms against one another at this time. The trial’s design specifies parameters for these intermediate analyses separately from the final analyses. The trial is designed to detect a clinically relevant benefit in the definitive primary outcome measure - overall survival. The assumption is that any such benefit should be preceded by a relative benefit at least as large in the intermediate primary outcome measure - failure-free survival (FFS). There is high power for each pairwise comparison throughout (≥90%) the trial and the pairwise significance level moves from permissive at the intermediate stages to strict at the final stage. Activity cut-point guidelines are derived for each intermediate analysis using the methods of Royston *et al.*[1] Table 1 depicts the design parameters. These are achieved using the n stage program, available in Stata [8,9].

**Table 1:** Design parameters by trial stage for original research arms

**Trial Stage**	**Type**	**Primary outcome measure**	**Target hazard ratio**	**Power**	**One-sided Significance level**	**Critical HR**	**Trigger events**^**1**^**(control)**
1	Activity	FFS	0.75	95%	0.500	1.00	114
2	Activity	FFS	0.75	95%	0.250	0.92	215
3	Activity	FFS	0.75	95%	0.100	0.89	334
4	Efficacy	OS	0.75	90%	0.025	-	400
Overall	-	-	-	83%	0.013	-	-

^1^ Number of control arm events provoking analyses for the original research comparisons with a 2:1 allocation ratio in favour of control. The required number of events would be different for an equal allocation ratio.

**Key: ***FFS * = failure-free survival; *OS* = overall survival; *HR* = hazard ratio.

**Note:** these parameters are used for each pairwise comparison of research vs control.

The intermediate analyses are triggered when a set number of events have been observed in the control arm. Focusing on the control arm in this way enables each analysis to be monitored and anticipated without revealing data on accumulating differences (or a lack of them) from the research arms. By considering the observed accrual rate and accumulating event rate in the control arm, it is possible to anticipate the time of the next intermediate analysis with reasonable precision. This can be calculated with the ARTPEP program (also available in Stata) [10]. Therefore, the first step towards planning can easily be achieved, which facilitates the planning of when the Independent Data Monitoring Committee (IDMC) should meet to review the data.

A large proportion of agents are unsuccessful in phase III trials [11-13], so it can be reasonably anticipated that accrual to at least one research arm in a MAMS trial is likely to be stopped early. Therefore, actions can, and should be, planned in advance of any early stopping decision. The statistical design explicitly considers stopping for lack-of-benefit, but research arms can also be stopped for reasons of harm or toxicity. Plans for either contingency can therefore be developed in anticipation of either eventuality.

### Advanced preparation

In the governance and oversight structure of STAMPEDE, the IDMC is advisory to a Trial Steering Committee (TSC), which has independent members (including the Chair) and representatives from the Trial Management Group (TMG), the latter being the committee responsible for the day-to-day running of the trial. The IDMC decides which accumulating data, if any, should be seen by the TSC executive when they next meet and also guides interpretation.

Before the first intermediate analysis, a joint meeting of the TSC and IDMC was held in which the design of the trial was reviewed and a number of hypothetical scenarios were considered, anticipating a number of different ‘results’ at the intermediate stages. The aim was to provoke discussion on what sort of decisions each committee might make when reviewing certain outcomes and what information the two committees could expect to see to make such recommendations and decisions.

A series of “what if…?” questions were considered months before the first intermediate activity analysis discussions were held within the trials unit and within the TMG about the actions and communications required before and after each intermediate analysis. These questions included the following: What if one arm was stopped for safety after a given review or was stopped for lack-of-benefit; what if more than one arm was halted and for the same or different reasons; and even, what if no changes were made?

Table 2 summarizes the planned actions and timelines targeted. Communicating to trial sites that changes might be forthcoming was started many weeks before each intermediate analysis. The potential for stopping recruitment to arms was built into the trial design; therefore, such activity did not require a formal protocol amendment. A letter was circulated to each clinical site from the regulatory authority confirming this fact.

**Table 2:** Planned actions and timelines when accrual is stopped to trial arms

**Action required**	**Timelines**	
	**Safety**	**LOB**
Notify sites in writing of IDMC meeting date to pre-warn	-28 d	-28 d
Circulate prior letter from regulatory agency confirming that stopping early for LOB is not a substantial amendment, but part of trial design	-28 d	-28 d
IDMC meeting	-7 d	-7 d
IDMC notes and recommendations finalised	(<1 w)	(<1 w)
TSC meeting: stop / continue decision for each research arm	Day 0	Day 0
Turn off randomisation to arms stopping early for safety	<24 h	<1 w
Notify centres by email; patients to ignore irrelevant parts of PIS	<24 h	<24 h
Notify relevant industry partners	<24 h	<24 h
Notify TMG members	<24 h	<24 h
Alert trials unit staff to potential queries	<24 h	<48 h
Phone all site PIs. Instructed to hand-amend PIS and CF. Updated documentation to follow	<1 w	<1 w
Protocol and documents updated and agreed by TMG	<1 w	<1 w
Summary information for patients	<2 w	<2 w
Notify ethics committee and regulatory agency (for information only)	<2 w	<2 w
Detailed discussions with industry partners	<1 m	<1 m
TMG review of processes	<1 m	<1 m

**Key: ***LOB * = lack-of-benefit; *IDMC* = Independent Data Monitoring Committee; *TSC* = Trial Steering Committee; *TMG* = Trial Management Group; *PIS* = patient information sheet; *CF* = consent form; *h* = hour; *w* = week.

**Note:** the observed timelines broadly followed these plans.

Many activities (Table 2) were planned to occur quickly after a TSC decision to stop recruitment to any arm. The planned timelines were the same for most activities, regardless whether stopping an arm for lack-of-benefit or toxicity; although the plans for notifying staff internally and turning off randomisation needed to be quicker in the instance of toxicity. The reason for this disparity in times is because it was anticipated that arms showing a lack-of-benefit could actually be showing a trend towards an advantage to the research arm, albeit an insufficiently large advantage to encourage recruitment of more patients to that comparison. For example, at the third intermediate analysis, the activity cut point is a hazard ratio of 0.89. Therefore, accrual may be stopped to an arm with a favorable hazard ratio of 0.90. In the absence of an overriding safety concern it was reasonable to allow a little more time to turn off allocation to such a treatment.

Notifications to the ethics committee(s) and the regulators were planned. Although no immediate protocol amendment would be required, it was decided that the protocol would be updated as soon as practicable after changes to study arms to simplify activities for sites. The Patient Information Sheets (PIS) would be updated immediately with removal of information that was no longer pertinent and sent to the ethics committee(s) for information.

### Results

The IDMC has met at least annually to review accumulating data. The data for the first formal intermediate activity analysis were frozen on 09 February 2010 with 1,424 patients randomized and 129 control-arm FFS events. The HR guideline cut point was 1.00. The review took place on 30 March 2010 by which time 1,518 patients had been randomized. The IDMC reviewed all data on the main outcome measures and recommended no changes.

The data for the second formal intermediate activity analysis were frozen on 01 February 2011 with 2,043 patients randomized and 209 control arm FFS events. The HR cut point was now 0.92. The IDMC meeting took place on 31 Mar 2011 by which time 2,163 patients had been randomized. The IDMC recommended that (i) the TSC see FFS data for both arms containing celecoxib and (ii) that no further patients be recruited to either celecoxib-containing arm. The detailed clinical findings are reported elsewhere [14,15].

### Practical experience

The TSC met on 06 April 2011 and approved the IDMC recommendations. They decided that (i) accrual should stop to both celecoxib-containing arms (D and F), following the lack-of-benefit guidelines; (ii) patients currently receiving celecoxib should be advised to stop celecoxib; (iii) comparative data celecoxib *versus* control arm (D *versus* A) should be shared with investigators; and (iv) data from the celecoxib plus zoledronic acid arm (F) should not be released as recruitment to the other zoledronic acid-containing arms (B and E) was to continue.

The observed HR for celecoxib *versus* control was 0.98, which did not meet the cut point. Although there was no clear evidence of either harm or unacceptable toxicity, no drugs are without potential for unwanted side-effects, and there was insufficient evidence of benefit to outweigh such risk.

As some patients were being advised to stop their treatment, it was decided to follow the safety rather than the lack-of-benefit timelines. By the afternoon of Day 0, (TSC meeting day) on 06 April 2011, the allocation to the celecoxib-containing arms in the randomization system had been disabled. All sites were notified by email that day and were told to re-advise patients who had consented to the trial but who had not yet been randomized. The Chief Investigator, patient representative and trials unit also agreed to a temporary revised version of the PIS. A quick response to this was facilitated by drafting many versions of the PIS before the IDMC meeting to reflect the needs following a number of hypothetical outcomes. Sites were offered a new electronic version of the revised PIS or they could manually cross out the few newly irrelevant sections of their current version. Sites were also told to advise patients to stop taking celecoxib at their next routine visit but that no urgent visits by patients to the clinic were required. A small number of patients who had been on celecoxib for many months chose to remain on treatment; only five patients are reported as still being on celecoxib at the start of July 2011.

On Day 1, phone calls were made to all site principal investigators (PIs) to ensure they were aware of and understood the actions taken. The trial team also worked with consumers to develop a summary of the results and actions to give to all patients. We thought it important for all trial patients to understand this information, not just those patients allocated to celecoxib-arms. This summary of results and actions was provided to sites for distribution to patients. Finally, industrial partners were apprised of the actions.

By Day 7, the PIS had been formally updated, submitted to the main ethics committee for information and distributed to sites. The regulatory authority was formally notified at this time. The protocol was formally amended (to version 7.0) after two months and submitted in June 2011 to the regulatory authority and ethics committee as a non-substantial amendment for information only.

These processes ran smoothly and no problems were reported from any of the governance bodies or from participating sites. The most common question from a small number of sites related to the use of new documents without apparent ‘prior’ approval. The earlier letter from the regulatory agency was sufficient to respond to these queries.

Recruitment continued to the control arm and to the remaining three research arms at good rates, with 47 patients in the 4 full weeks prior to change and 42 patients in the subsequent 4 full weeks. Importantly, follow-up continues for patients allocated to the celecoxib-containing arms.

### Patients recently randomized to celecoxib-containing arms

A small number of patients randomized shortly prior to the halting of the celecoxib-containing arms had not necessarily had a chance to start their allocated treatment. It was agreed that patients allocated to arm D (HT plus celecoxib) and who still met all of the eligibility criteria for new patients joining the trial could be offered the opportunity to withdraw from STAMPEDE and to be re-randomized as a new patient. This was discussed with the relevant sites and five patients of seven meeting these criteria chose re-randomization. Since recruitment to and treatment within arm B (zoledronic acid) was continuing, patients on arm F (celecoxib plus zoledronic acid) were recommended to stop their celecoxib treatment and were not offered re-randomization. No patient will contribute more than once to any comparison. Sensitivity analyses will be considered.

### Handling investigator assumptions

Investigators will no doubt make implicit assumptions about any research arms that continue accrual beyond each intermediate analysis. However, community uncertainty should not be affected by unreleased data from these intermediate comparisons. The knowledge that there are likely encouraging data on an early outcome measure at the intermediate analyses just reinforces the need to continue randomization to gain stronger evidence for the definitive primary outcome measure. If any of the arms showed early results for the definitive primary outcome measure that would be sufficiently convincing to influence clinical practice, the IDMC could recommend early release of the data. The strengths and weaknesses of that data would be discussed.

## Designing new comparisons

### Rationale for including new research arms

The practical reasons for choosing the five research arms at the outset of STAMPEDE has been discussed previously [7]. The STAMPEDE TMG has now successful activated one further research arm (G, abiraterone) and further arms are being considered.

The motivations for including a new research arm are many. The philosophy at the outset was to evaluate multiple agents from different classes but as new agents emerge and potential synergies from agents within the same class become a possibility, it is important that consideration is given to testing these alone, and possibly in combination, within the framework of a MAMS trial design. Agents and approaches should only be considered for addition when there are robust and convincing scientific hypotheses.

There is huge interest in new ‘hormone therapies’ for prostate cancer with the first such agent, abiraterone, an androgen biosynthesis inhibitor, licensed in 2011. Drugs approved in patients with late stage disease will generally go on to be tested in earlier settings. The efficiencies available in MAMS trials [3] are as relevant to new agents as they are in the original arms. These efficiencies include the following: (i) using one protocol to allow for new comparisons to be introduced by amendment; (ii) quicker recruitment to the new comparison by activating participating sites for the new comparison more quickly than is possible in any new, separate study; (iii) the increased chance of allocation of a research arm, which may appeal to many patients, thereby boosting accrual; (iv) the contemporaneous accrual to comparisons which overlap in time means no gaps between, and no competition between, trials; (v) phase II assessments of activity are incorporated; and (vi) together, the duration and cost of such a setting up a new comparison in this way would be considerably shorter and lower than any separate, new trial.

The rationale for considering any new research arm would need to be clear. The STAMPEDE TMG set forward a number of criteria summarized in Table 3.

**Table 3:** Criteria for potential new research arms


·	Sound rationale, including a robust biological hypothesis and compelling evidence of activity that strongly identifies a need to assess a research approach in the setting studied.
·	Positive evidence of mechanisms or synergy of action (or both) in the disease area
·	Investigator enthusiasm for the new research arm.
·	Pharmaceutical research agents would need to be licensed or be close to being licensed at the time of activation. Without a licensed use for the drug (usually in later disease), data in the target setting are likely to be of limited value.
·	Relevant industry partners willing to collaborate and contribute to the trial, if the research arm is a pharmaceutical agent.
·	Successful independent peer review as for a new study.
·	Recruitment to new arm must not jeopardise completion of the ongoing research arms, e.g. by diluting recruitment excessively. This means that the accrual rate must be better than predicted in the original trial or that other research arms have already stopped accrual early. STAMPEDE is presently recruiting at around 700 patients/year when 500 patients/year were targeted. This permitted capacity to consider new research agents even while all original arms remained open.
·	The new comparison must still be relevant when it matures.

### When to add new arms

Many new agents are being developed in prostate cancer, often with differing mechanisms of action and demonstrable survival advantages in the later, castrate-refractory stages of the disease. These include approaches based on cytotoxic chemotherapy (cabazitaxel) [16], immunotherapy (sipuleucel-T) [17], radionuclides (radium-223) [18], novel androgen blockade (MDV3100) [19-21], and cellular pathway targeting drugs (cabozantinib) [22]. However, ahead of all of these in the development process has been abiraterone acetate (Zytiga: Janssen Pharmaceuticals). HT eventually fails for most men, even though they have castrate levels of circulating androgens. This is called castration-refractory prostate cancer (CRPC). Recent evidence suggests the failure is due to persistent activity of the androgen receptor, occurring because of intracellular conversion of steroids precursors to androgenic steroids by prostate cancer cells. A key enzyme in this process is CYP17, an enzyme which represents a logical target for therapy in later stages of prostate cancer [23]. Abiraterone is a selective inhibitor of CYP17 and is highly active in patients developing resistance to standard androgen ablation therapies [24-26]. First results of a phase III study comparing abiraterone to placebo in CRPC patients following chemotherapy with docetaxel reported an absolute improvement in overall survival of more than 4 months, hazard ratio 0.65 (95% CI, 0.54 to −0.77) [27]. The drug received marketing authorization in European Union from September 2011 and there is a robust biological hypothesis [28]. The TMG hypothesized that abiraterone could have a greater absolute effect when given to men with high-risk disease starting long-term HT (that is, earlier in the time course of the disease and synchronously with standard androgen ablation).

### Design issues

The addition of HT plus abiraterone means that STAMPEDE has become a five-arm trial from mid-November 2011. The design parameters for STAMPEDE in Table 1 relate to each pairwise comparison of research arm against control arm. The same parameters and targets for the new comparison of abiraterone *versus* control have been chosen; these will only be made within contemporaneously recruited patients. That is, patients allocated to a new research arm are compared against control arm patients randomized after a new research arm was added.

Inevitably, analyses of new comparisons will be later than for the original comparisons. It is anticipated that completing accrual to the new research comparison (and possibly further additional arms) may occur before the primary survival results from the initial comparisons are known.

The key question was how many patients are required to attain these parameters? This depends on traditional and MAMS-specific factors, including recruitment rate, power and significance level for each stage. We realized that accrual would start strongly because the new comparison would quickly be activated in many sites. Furthermore, accrual to the new comparison would be boosted when recruitment to the original research arms stopped. A survey of participating sites (unpublished) suggested that overall accrual could even be boosted by including an ‘attractive’ new research arm. Accrual rates have increased throughout the trial (Figure 2) and could continue to increase regardless of the addition of a new arm.

For the sample size calculations, this comparison was first treated as if it were a two-arm multi-stage trial using nstage [8,9] to obtain the control arm events required to trigger the end of each stage. The duration of the trial was then estimated using ARTPEP [10] to estimate when these events would be attained. ARTPEP offers much flexibility in accrual rates, follow-up duration and loss-to-follow-up.

**Figure 3:**
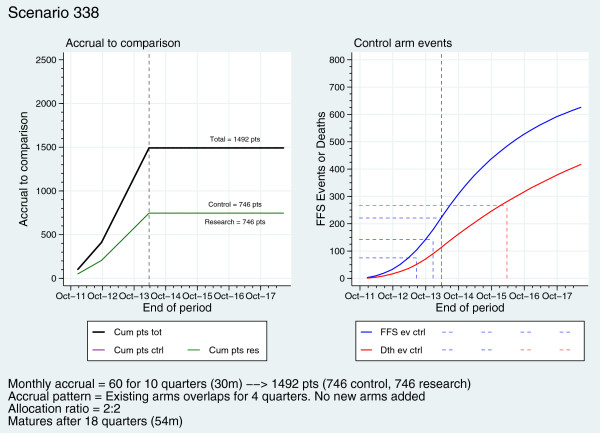
An example of accrual scenarios for the new comparison and project of cont rol arm events.

Figure 3 (left) shows projected cumulative accrual to the new comparison (not the full trial) in the basic scenario. A boost to the accrual rate for the comparison is seen when the original research arms complete accrual, here after 12 months of co-recruitment, a 2:2 allocation ratio is shown so the green and purple lines depicting accrual to each arm are overlaid. A 2:1 allocation ratio was chosen for the original comparisons. It is more efficient to have more patients allocated to the control arm when there are more research arms co-recruiting [7]. Accrual to the abiraterone comparison will continue after accrual to the original research arms completes. An equal allocation ratio is more efficient in a two-arm setting, bringing forward maturity by a few months. We further considered that a 2:1 allocation ratio favoring the control arm might negatively impact accrual and duration.

Figure 3 (right) shows cumulative FFS events and deaths expected on the control arm. The dashed lines mark the number of control events required to trigger the intermediate and final analyses and the approximate calendar time of these analyses (rounded to the nearest quarter-year) in this scenario. On both sides of the graph, the vertical, dashed, black line represents the end of accrual.

The TMG has constructed and stored many such scenarios for the original trial, including increasing or decreasing the accrual rate, the accrual period, the follow-up period, the length of co-recruitment with the original arms and the median FFS and overall survival times, as well as introducing one or two further research arms later in the trial.

Accrual to this comparison is planned to stop at the sooner of (i) 1,500 new patients allocated to control or abiraterone or (ii) three years after activating the abiraterone comparison. The comparison should mature in around five years, depending on the mix of metastatic and non-metastatic patients joining the trial. Importantly, accrual to this comparison should complete before the original comparisons mature for the definitive primary outcome measure.

### Practical experience

The abiraterone comparison was formally launched on Tuesday 15 November 2011. A total of 31 patients joined STAMPEDE by the end of the first full week, with 18 allocated to arms A or G (around the 4 of 7 expected). We describe the steps that we took to reach this point.

Janssen’s first formal results for abiraterone in late-stage prostate cancer were published in April 2011 [27] and updated at the American Society of Clinical Oncology (ASCO) 2011 [29], with abiraterone being licensed in Europe in September 2011. Discussions between the TMG and Janssen were formally initiated much earlier in March 2010 with an application by the trial team for an investigator-initiated study. In July 2010, the main funding body, Cancer Research United Kingdom, organized a scientific peer review of the revised trial application to conditionally amend STAMPEDE pending successful negotiations and a successful results from the Janssen’s COU-AA-301 trial. This included findings reflecting broad enthusiasm among investigators and patients to assess abiraterone, plus the TMG’s enthusiasm for this new approach.

Subsequent months were spent discussing the dose and duration of abiraterone, steroid support, details of drug supply and distribution, safety reporting, research supports and contracts with Janssen’s United Kingdom, European and Global affiliates. The sample size estimates were then finalized and the protocol was conditionally updated.

The principle of adding a new arm was discussed at each IDMC and TSC meeting in 2010 and 2011. The TSC reviewed the protocol in August 2011. The new research arm G (hormone therapy plus abiraterone) was then submitted to the regulatory agency and ethics committee late in August 2011 as a substantial protocol amendment (version 8.0) along with an updated PIS. This updated protocol included some modified eligibility criteria pertinent to assessing abiraterone. All patients now needed histological confirmation of prostate cancer, something not previously required for the subset of patients with bone metastases and PSA >100 ng/ml. In the United Kingdom this affected only a small proportion of patients and fitted in with current National Institute for Health and Clinical Excellence (NICE) guidance recommending a biopsy where a patient may wish to enter a trial. This is also consistent with most trials of new agents in prostate cancer. Further clarifications on liver and renal function that are associated with fitness for abiraterone were also introduced but were unlikely to markedly affect the number of eligible patients.

The TMG also introduced an unrelated change for non-metastatic patients following recent results from SPCG-7 [30] and MRC PR07/NCIC PR.3 [31], which demonstrated a survival advantage to HT plus radiotherapy over HT alone in men with locally advanced, non-metastatic prostate cancer. Previously, radiotherapy had been encouraged for such men in STAMPEDE, but the trial has been updated to reflect the new standard-of-care.

The likely addition of abiraterone had been extensively promoted at conferences and investigator meetings in addition to the survey. The trial team started training participating sites in parallel with the contractual and regulatory processes. Regional launch and training meetings in Glasgow, London, Cardiff and Manchester in September 2011 and October 2011 were supplemented with teleconferences. This included making clear that there would be a formal switchover date to protocol version 8.0 and that centers would need new local approval to maintain accrual beyond this date. That is, all sites needed to provide evidence to be given permission to start randomizing again.

The ethics committee responded quickly and positively in September 2011, the regulatory agency gave approval in October 2011 within their 35-day window, and neither body requested changes.

**Figure 4:**
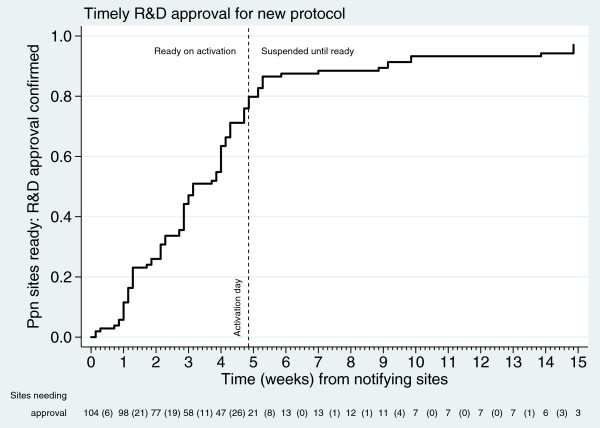
Activat ion of sites to new comparison.

The chosen date for the switchover was 15 November 2011, allowing sites 35 days from 12 October 2011 to attain local approvals. Figure 4 shows sites with local approvals for v8.0 over time. The first site gained approval within one week demonstrating the feasibility of quick amendments. By the time of final activation 83 of 104 sites had the necessary permissions in place. This denominator includes some sites that had not previously participated and were waiting for this protocol amendment. Four-fifths were ready within five weeks. This does not include centers in Switzerland, where the coordinating body, the Swiss Group for Clinical Cancer Research (Schweizerische Arbeitsgemeinschaft für Klinische Krebsforschung or SAKK), chose not to submit for approvals until after activation in the United Kingdom. All Swiss sites suspended accrual. Sites that were slower in activating include three that shared one R&D department, one where the PI had recently changed and one with unrelated pharmacy problems. The trial team ensured that all sites with approvals prior to switchover had access to abiraterone on-site from the day of activation.

It is unlikely that any completely new trial could have 83 sites ready to recruit on activation day, something that would be a considerable achievement. Figure 2 shows (on the left axis) the number of sites recruiting their first patient to the trial, demonstrating how long *de novo* trials may take to activate sites. Site activation was deliberately limited during the original Pilot Phase, but new sites have continued to activate throughout, giving accelerated recruitment rates. Therefore, a new, separate trial of abiraterone would likely have taken much longer to get to the same rate of recruitment as STAMPEDE achieved immediately. The cost of a separate trial would also have been higher if the trial took longer to conduct. A separate trial would also potentially have become established as a competing trial, with the possibility of an adverse effect on recruitment to both trials. Combining the new comparison into STAMPEDE also brings efficiencies in costs regarding site training, site monitoring and database construction.

**Figure 5:**
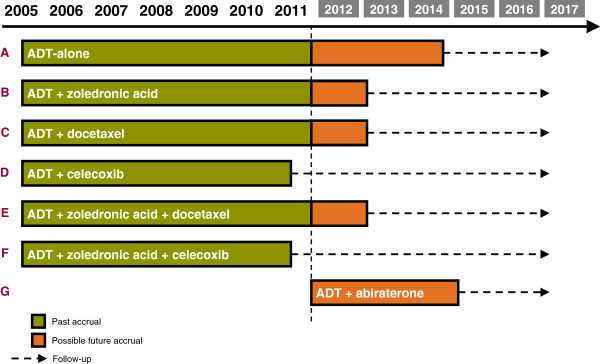
Trial arms open to allocat ion and fur ther t imelines as of Nov 2011.

We anticipated that accrual would be ahead of the switchover with sites that might want to wait until after switchover to randomize new patients. However, in the last full week prior to switchover 18 patients were randomized and 4 further patients were randomized the day before switchover. On the day of activation, 15 November 2011, 6 patients were randomized and by the end of January 2011, 53 patients were allocated to arm G. Figure 5 summarizes the current design of the trial and projected timelines.

### Other issues

Randomization is achieved through minimization with a random element [32,33] where 80% patients are allocated to a minimizing arm across a series of clinically-relevant stratification factors [7]. Therefore, at any moment in time, the groups allocated to each arm are well-balanced. After consideration, the TMG wiped the stratification tables clean when the new comparison was activated, effectively starting over again.

The possibility of the original research arms maturing while the new comparison was ongoing was discussed. Such data can be considered as if they are data emerging from an external trial. Indeed, there are some other trials which overlap partially with STAMPEDE in terms of both population and treatment. The trial team would react if change is needed, as demonstrated by the introduction of radiotherapy in non-metastatic disease.

### The future

The STAMPEDE investigators appreciate that challenging and novel changes can only be made by working with enthusiastic researchers. The group has, therefore, encouraged discussions with sites from the outset and has repeatedly engaged sites to ensure clinician and patient input and ‘buy-in’. The potential changes have been presented in newsletters and at regional meetings over a long period. Furthermore, researchers have been encouraged to bring forward ideas for further arms that might be worthy of assessment in this trial population.

Indeed, the STAMPEDE TMG has started to consider further arms. In September 2011 the TMG prioritized potential questions. Any new comparison would take at least one year to develop and activate so it is important that such discussions start early if they are to be activated. Champions for potential new agents have been making contact with potential industry partners although the most favored question was radiotherapy for metastatic disease, which pertains only to a subgroup of patients. This provides a further challenge for our flexible design to address. Projections for various recruitment scenarios demonstrate that this could be achieved if participating centers are keen to formulate questions together. A questionnaire was distributed to sites in November 2011 and the concept was received positively. This has been approved by the trial committees and has been successfully peer-reviewed by Cancer Research United Kingdom. A formal amendment to activate this comparison will be developed during summer 2012. We estimated that undertaking this comparison within STAMPEDE would cost only about 60% as much as a separate stand-alone trial.

## Conclusions

STAMPEDE is a high-profile adaptive randomized controlled trial (RCT). We believe the merits in this flexible approach are clearly apparent, and we encourage others to implement this or similar designs in other settings. In particular, the considerable costs associated with trial start up and close down can be substantially reduced by evaluating many new treatments simultaneously and also by using intermediate analyses to run a seamless phase II/III approach [3]. The ability to rapidly add new arms is also an important innovation. We have essentially set up a new phase III comparison of abiraterone in 100 centers in 18 months from the first idea to first patient; From protocol sign-off to opening was less than 4 months. The price in trial administration costs (ignoring drug associated costs which accrue whatever route one chooses) ran into tens of thousands of pounds sterling rather than the much larger sums normally associated with trial set up on this scale *de novo*. Experience shows that funders, regulators, investigators and, most importantly, patients can deal with the complex issues involved. Indeed, as the trial has progressed, companies wishing to have their drug included within the trial have started to approach us.

We hope that this very positive experience of an adaptive trial will encourage others to undertake further trials like STAMPEDE and that our experience will smooth the passage of further such trials.

In summary, the STAMPEDE trial has confirmed the notion that MAMS trials speed the evaluation of new treatments by testing many treatments simultaneously and by using lack-of-benefit analyses to focus research efforts towards the more promising research arms. It has also demonstrated that accrual and treatment of insufficiently active arms can be stopped successfully without this radical change affecting the continued evaluation of agents still undergoing testing in this setting. Finally, and importantly, we have demonstrated that adding additional research arms to enable new and rapid comparisons of novel agents either alone or in combination is possible, and that the modification of a complex protocol to accommodate changes in practice during the running of a MAMS study is achievable. This is particularly important in an ongoing trial of this type. We have shown that a major change to the protocol can be swiftly and effectively achieved in large numbers of centers, and high volume recruitment can be continued in a seamless, safe and efficient manner.

## Abbreviations

ADT: Androgen deprivation therapy; AS: Activity Stage; ASCO: American Society of Clinical Oncology; CRPC: castration-refractory prostate cancer; CTA: Clinical Trials Authorization; CTAAC: Clinical Trials Advisory and Award Committee; CTU: MRC Clinical Trials Unit; FFS: Failure-free survival; HR: Hazard ratio; HT: Hormone therapy; IDMC: Independent Data Monitoring Committee; MAMS: Multi-Arm Multi-Stage; MRC: Medical Research Council; NICE: National Institute for Health and Clinical Excellence; OM: Outcome measure; OS: Overall survival; PI: Principal Investigator; PIS: Patient Information Sheet; PSA: Prostate-specific antigen; RCT: Randomized Controlled Trial; R&D: Research and development; SAKK: Swiss Group for Clinical Cancer Research (Schweizerische Arbeitsgemeinschaft für Klinische Krebsforschung); STAMPEDE: Systemic Therapy for Advanced or Metastatic Prostate cancer: Evaluation of Drug Efficacy; TMG: Trial Management Group; TSC: Trial Steering Committee.

## Authors’ contributions

MRS, trial statistician, TMG member and grant holder, designed the trial, collated data, performed analyses, interpreted data, wrote critical sections of manuscript, and developed and implemented methods. NDJ, chief investigator, TMG chair and grant holder, designed the trial, collated data, interpreted data, wrote critical sections of manuscript, and developed and implemented methods. MDM, TMG vice-chair and grant holder, designed the trial, and collated and interpreted data. NWC, TMG vice-chair and grant holder, designed the trial, and collated and interpreted data. CA, TMG member, coordinated the trial, interpreted data, and developed and implemented methods. JA, TMG member, collated and interpreted data. JdB, TMG member and grant holder, designed the trial, and collated and interpreted data. DPD, TMG member and grant holder, designed the trial, and collated and interpreted data. JD, TMG member, designed the trial and interpreted data. CG, TMG member, coordinated the trial, and developed and implemented methods. GJ, statistician and TMG member, collated data, performed analyses, and interpreted data. AR, trial surgeon and TMG member, interpreted data, developed and implemented methods, and performed clinical review of pharmacovigilance data. JMR, TMG member, collated and interpreted data. KS, TMG member, coordinated the trial, collated and interpreted data, and developed and implemented methods. GT, Swiss national coordinator and TMG member, collated and interpreted data. MKBP, TMG member and grant holder, designed the trial, interpreted data, developed and implemented methods. All authors read and approved final manuscript.

## Competing interests

Matthew R Sydes, Claire Amos, Charlene Green, Gordana Jovic, Alastair Ritchie and Karen Sanders are employees of the Medical Research Council. Mahesh Parmar is an employee of the Medical Research Council and has Honoraria for IDMC membership from Sanofi-Aventis. Johann de Bono is employed by the Institute of Cancer Research which has a financial interest in abiraterone and has served on advisory boards for Sanofi-Aventis, Novartis, Pfizer, Janssen. Nick James has served on advisory boards for Sanofi-Aventis, Novartis, Pfizer. David Dearnaley is employed by the Institute of Cancer Research which has a financial interest in abiraterone and has served on advisory boards for Novartis. Malcolm Mason has honoraria and expenses for serving on advisory boards and lecturing for Sanofi-Aventis. Noel W Clarke, John Anderson, John Dwyer, Martin Russell and George Thalmann have no interests to declare.

## Disclosure

This manuscript is based on an oral presentation given at the first United Kingdom Clinical Trials Methodology Conference, Bristol, UK in October 2011 and subsequently presented as a poster as the Genitourinary Cancer Symposium, San Francisco, USA in February 2012.

## References

[B1] Royston P Parmar MKB Qian W Novel designs for multi-arm clinical trials with survival outcomes with an application in ovarian cancer Stat Med 2003 22 2239 2256 10.1002/sim.1430 12854091

[B2] Royston P Barthel FMS Parmar MKB Choodari-Oskooei B Isham V Designs for clinical trials with time-to-event outcomes based on stopping guidelines for lack of benefit Trials 2011 12 81 10.1186/1745-6215-12-81 21418571 PMC3078872

[B3] Parmar MKB Barthel F Sydes M Langley R Kaplan R Eisenhauer E Brady M James N Bookman MA Swart AM Qian W Royston P Speeding up the evaluation of New agents in cancer J Natl Canc Inst 2008 100 1204 1214 10.1093/jnci/djn267 PMC2528020 18728279

[B4] James N Sydes M Clarke N Mason M Dearnaley D Anderson J Popert R Sanders K Morgan R Stansfeld J Dwyer J Masters J Parmar MKB STAMPEDE: systemic therapy for advancing or metastatic prostate cancer - a multi-Arm multi-stage randomised controlled trial Clin Onc 2008 20 577 581 10.1016/j.clon.2008.07.002 18760574

[B5] James ND Sydes MR Clarke NW Mason MD Dearnaley DP Anderson J Popert RJ Sanders K Morgan RC Stansfeld J Dwyer J Masters J Parmar MKB Systemic therapy for advancing or metastatic prostate cancer (STAMPEDE): a multi-arm, multistage randomized controlled trial BJU Int 2009 103 464 469 10.1111/j.1464-410X.2008.08034.x 18990168

[B6] http://www.stampedetrial.org

[B7] Sydes MR Parmar MKB James ND Clarke NW Dearnaley DP Mason MD Morgan RC Sanders K Royston P Issues in applying multi-arm multi-stage methodology to a clinical trial in prostate cancer: the MRC STAMPEDE trial Trials 2009 10 39 10.1186/1745-6215-10-39 19519885 PMC2704188

[B8] Barthel FM-S Royston P Babiker A A menu-driven facility for complex sample size calculation in randomized controlled trials with a survival or a binary outcome: Update Stata J 2005 5 123 129

[B9] Barthel FM-S Royston P Parmar MKB A menu-driven facility for sample-size calculation in novel multiarm, multistage randomized controlled trials with a time-to-event outcome Stata J 2009 9 505

[B10] Royston P Barthel FM Projection of power and events in clinical trials with a time-to-event outcome Stata J 2010 10 1 9

[B11] Kola I Landis JR Can the pharmaceutical industry reduce attrition rates? Nat Rev Drug Discov 2004 3 711 715 10.1038/nrd1470 15286737

[B12] Kumar A Soares H Wells R Clarke M Hozo I Bleyer A Reaman G Chalmers I Djulbegovic B Are experimental treatments for cancer in children superior to established treatments? Observational study of randomised controlled trials by the Children's Oncology Group. BMJ 2005 331 1295 10.1136/bmj.38628.561123.7C PMC1298846 16299015

[B13] Roberts TG Jr Lynch TJ Jr Chabner BA The phase III trial in the era of targeted therapy: unraveling the "go or no go" decision J Clin Oncol 2003 21 3683 3695 10.1200/JCO.2003.01.204 14512401

[B14] James ND Sydes MR Mason MD Clarke NW Anderson J Dearnaley DP Dwyer J Jovic G Ritchie AWS Russell JM Sanders K Thalmann GN Bertelli G Birtle AJ O'Sullivan JM Protheroe A Sheehan D Srihari N Parmar MKB Celecoxib plus hormone therapy versus hormone therapy alone for hormone-sensitive prostate cancer: first results from the STAMPEDE multiarm, multistage, randomised controlled trial Lancet Oncol 2012 13 549 558 10.1016/S1470-2045(12)70088-8 22452894 PMC3398767

[B15] James ND Sydes MR Mason MD Clarke NW Dearnaley DP Dwyer J Jovic G Russell JM Thalmann G Parmar MKB Celecoxib plus hormone therapy Vs hormone therapy alone for hormone-sensitive prostate cancer: first results from the STAMPEDE randomised controlled trial (MRC PR08) Eur J Cancer 2011 Suppl 2 11

[B16] de Bono JS Oudard S Ozguroglu M Hansen S Machiels JP Kocak I Gravis G Bodrogi I Mackenzie MJ Shen L Roessner M Gupta S Sartor AO Prednisone plus cabazitaxel or mitoxantrone for metastatic castration-resistant prostate cancer progressing after docetaxel treatment: a randomised open-label trial Lancet 2010 376 1147 1154 10.1016/S0140-6736(10)61389-X 20888992

[B17] Kantoff PW Higano CS Shore ND Berger ER Small EJ Penson DF Redfern CH Ferrari AC Dreicer R Sims RB Xu Y Frohlich MW Schellhammer PF Sipuleucel-T immunotherapy for castration-resistant prostate cancer N Engl J Med 2010 363 411 422 10.1056/NEJMoa1001294 20818862

[B18] Nilsson S Franzen L Parker C Tyrrell C Blom R Tennvall J Lennernas B Petersson U Johannessen DC Sokal M Pigott K Yachnin J Garkavij M Strang P Harmenberg J Bolstad B Bruland OS Bone-targeted radium-223 in symptomatic, hormone-refractory prostate cancer: a randomised, multicentre, placebo-controlled phase II study Lancet Oncol 2007 8 587 594 10.1016/S1470-2045(07)70147-X 17544845

[B19] Scher HI Beer TM Higano CS Anand A Taplin ME Efstathiou E Rathkopf D Shelkey J Yu EY Alumkal J Hung D Hirmand M Seely L Morris MJ Danila DC Humm J Larson S Fleisher M Sawyers CL Antitumour activity of MDV3100 in castration-resistant prostate cancer: a phase 1–2 study Lancet 2010 375 1437 1446 10.1016/S0140-6736(10)60172-9 20398925 PMC2948179

[B20] Tran C Ouk S Clegg NJ Chen Y Watson PA Arora V Wongvipat J Smith-Jones PM Yoo D Kwon A Wasielewska T Welsbie D Chen CD Higano CS Beer TM Hung DT Scher HI Jung ME Sawyers CL Development of a second-generation antiandrogen for treatment of advanced prostate cancer Science 2009 324 787 790 10.1126/science.1168175 19359544 PMC2981508

[B21] Scher HI Fizazi K Saad F Taplin M-E Sternberg CN Miller MDK Increased Survival with Enzalutamide in Prostate Cancer after Chemotherapy New Engl J Med 2012 Published online 15-Aug-2012 10.1056/NEJMoa1207506 22894553

[B22] Hussain M Smith MR Sweeney C Corn PG Elfiky A Gordon MS Haas NB Harzstark AL Kurzrock R Lara P Lin C Sella A Small EJ Spira AI Vaishampayan UN Vogelzang NJ Scheffold C Ballinger MD Schimmoller F Smith DC Cabozantinib (XL184) in metastatic castration-resistant prostate cancer (mCRPC): results from a phase II randomized discontinuation trial J Clin Oncol 2011 29 4516 10.1200/JCO.2010.33.7741 22025164

[B23] Attard G Richards J de Bono JS New strategies in metastatic prostate cancer: targeting the androgen receptor signaling pathway Clin Cancer Res 2011 17 1649 1657 10.1158/1078-0432.CCR-10-0567 21372223 PMC3513706

[B24] Danila DC Morris MJ de Bono JS Ryan CJ Denmeade SR Smith MR Taplin ME Bubley GJ Kheoh T Haqq C Molina A Anand A Koscuiszka M Larson SM Schwartz LH Fleisher M Scher HI Phase II multicenter study of abiraterone acetate plus prednisone therapy in patients with docetaxel-treated castration-resistant prostate cancer J Clin Oncol 2010 28 1496 1501 10.1200/JCO.2009.25.9259 20159814 PMC3040042

[B25] Reid AH Attard G Danila DC Oommen NB Olmos D Fong PC Molife LR Hunt J Messiou C Parker C Dearnaley D Swennenhuis JF Terstappen LW Lee G Kheoh T Molina A Ryan CJ Small E Scher HI de Bono JS Significant and sustained antitumor activity in post-docetaxel, castration-resistant prostate cancer with the CYP17 inhibitor abiraterone acetate J Clin Oncol 2010 28 1489 1495 10.1200/JCO.2009.24.6819 20159823 PMC2849770

[B26] Ryan CJ Smith MR Fong L Rosenberg JE Kantoff P Raynaud F Martins V Lee G Kheoh T Kim J Molina A Small EJ Phase I clinical trial of the CYP17 inhibitor abiraterone acetate demonstrating clinical activity in patients with castration-resistant prostate cancer who received prior ketoconazole therapy J Clin Oncol 2010 28 1481 1488 10.1200/JCO.2009.24.1281 20159824 PMC2849769

[B27] de Bono JS Logothetis CJ Molina A Fizazi K North S Chu L Chi KN Jones RJ Goodman OB Jr Saad F Staffurth JN Mainwaring P Harland S Flaig TW Hutson TE Cheng T Patterson H Hainsworth JD Ryan CJ Sternberg CN Ellard SL Flechon A Saleh M Scholz M Efstathiou E Zivi A Bianchini D Loriot Y Chieffo N Kheoh T Hagg CM Scher HI COU-AA-301 Investigators COU-AA-301 Investigators Abiraterone and increased survival in metastatic prostate cancer N Engl J Med 2011 364 1995 2005 10.1056/NEJMoa1014618 21612468 PMC3471149

[B28] de Bono JS Ashworth A Translating cancer research into targeted therapeutics Nature 2010 467 543 549 10.1038/nature09339 20882008

[B29] Group obotC-A-S Scher HI Logothetis C Molina A Goodman OB Sternberg CN Chi KN Kheoh TS Haqq CM Fizazi K Bono JSD Improved survival outcomes in clinically relevant patient subgroups from COU-AA-301, a phase III study of abiraterone acetate (AA) plus prednisone (P) in patients with metastatic castration-resistant prostate cancer (mCRPC) progressing after docetaxel-based chemotherapy J Clin Oncol 2011 29 4 10.1200/JCO.2010.32.1752 21115867

[B30] Widmark A Klepp O Solberg A Damber JE Angelsen A Fransson P Lund JA Tasdemir I Hoyer M Wiklund F Fossa SD Endocrine treatment, with or without radiotherapy, in locally advanced prostate cancer (SPCG-7/SFUO-3): an open randomised phase III trial Lancet 2009 373 301 308 10.1016/S0140-6736(08)61815-2 19091394

[B31] Warde P Mason M Ding K Kirkbride P Brundage M Cowan R Gospodarowicz M Sanders K Kostashuk E Swanson G Barber J Hiltz A Parmar MKB Sathya J Anderson J Hayter C Hetherington J Sydes MR Parulekar W for the NCIC CTG PR.3/MRC UK PR07 investigators for the NCIC CTG PR.3/MRC UK PR07 investigators Combined androgen deprivation therapy and radiation therapy for locally advanced prostate cancer: a randomised, phase 3 trial Lancet 2011 378 2104 2111 10.1016/S0140-6736(11)61095-7 22056152 PMC3243932

[B32] Pocock S Clinical Trials: A Practical Approach 1983 New York: Wiley

[B33] Taves DR Minimization: a new method of assigning patients to treatment and control groups Clin Pharmacol Ther 1974 15 443 453 4597226 10.1002/cpt1974155443

